# Sensitizing events as trigger for discursive renewal and institutional change in Flanders’ environmental health approach, 1970s-1990s

**DOI:** 10.1186/1476-069X-12-46

**Published:** 2013-06-07

**Authors:** Kristien R Stassen, Roel Smolders, Pieter Leroy

**Affiliations:** 1 HUB – Hogeschool-Universiteit Brussel, Centre for Corporate Sustainability, Warmoesberg 26, Brussels, 1000, Belgium; 2 VITO – Flemish Institute for Technological Research, Unit Environment and Health, Boeretang 200, Mol, 2400, Belgium; 3 Institute for Management Research, Political Sciences of the Environment, Radboud University, Nijmegen, P.O. Box 9108, Nijmegen, HK, 6500, the Netherlands

**Keywords:** Discursive institutionalism, Environmental health incidents, Policy arrangement approach

## Abstract

**Background:**

Sensitizing events may trigger and stimulate discursive renewal. From a discursive institutional perspective, changing discourses are the driving force behind the institutional dynamics of policy domains. Theoretically informed by discursive institutionalism, this article assesses the impact of a series of four sensitizing events that triggered serious environmental health concerns in Flanders between the 1970s till the 1990s, and led onto the gradual institutionalization of a Flemish environmental health arrangement.

**Methods:**

The Policy Arrangement Approach is used as the analytical framework to structure the empirical results of the historical analysis based on document analysis and in-depth interviews.

**Results:**

Until the 1990s, environmental health was characterized as an ad hoc policy field in Flanders, where agenda setting was based on sensitizing events – also referred to as incident-driven. Each of these events contributed to a gradual rethinking of the epistemological discourses about environmental health risks and uncertainties. These new discourses were the driving forces behind institutional dynamics as they gradually resulted in an increased need for: 1) long-term, policy-oriented, interdisciplinary environmental health research; 2) policy coordination and integration between the environmental and public health policy fields; and 3) new forms of science-policy interactions based on mutual learning. These changes are desirable in order to detect environmental health problems as fast as possible, to react immediately and communicate appropriately.

**Conclusions:**

The series of four events that triggered serious environmental health concerns in Flanders provided the opportunity to rethink and re-organize the current affairs concerning environmental health and gradually resulted into the institutionalization of a Flemish environmental health arrangement.

## Background

Possible adverse health impacts of the deteriorating environmental quality have been an issue since the early days of environmental concern, as indicated in the Declaration of the United Nations Conference on the Human Environment in 1972. In the late 1980s, the issue regained attention by the Environment and Health Process for Europe, initiated by the World Health Organization Europe (WHO-Europe). Its five-yearly European Conferences on Environment and Health have played an important role in the agenda setting of the environmental health discourse at the European level. The main discourses relate to policy integration, stakeholder involvement, and children as a prioritized target group. As already concluded in our previous paper [1], these novel discourses have greatly affected the European environmental health policy arrangement in terms of the establishment of new organizations, agreements, charters, financial resources, and legislation. Yet, the implementation of European discourses and agreements unfolded rather slowly in Belgium, partly due to its internal complexity. The Belgian State is characterized by a federal state composed of three Regions and three Communities which have the authorities on environment and health prevention policies respectively. The previous study observed that local events that triggered serious environmental health concerns seem to have induced institutional change in Flanders, the northern region of Belgium. We analyze their role in full detail in this paper, well aware of the role of international and European developments and of similar events in other countries and regions.

The main objective of this contribution is to assess the impact of this series of four domestic events between the 1970s till the 1990s on the institutionalization of the Flemish environmental health arrangement, both scientifically and politically. Theoretically informed by discursive institutionalism, this article assumes that this series of events had gradually shifted the environmental health discourses of politicians, scientists, and the population in general. These shifting discourses, in turn, triggered the institutional renewal of the scientific knowledge-production process as well as the environmental health policy arrangement, by inducing and introducing novel organizational facilities, new methodological tools, policy programmes, budgets, rules, etc. [2].

This article structures as follows: the first section further positions sensitizing events as a trigger to discursive renewal, and the impact of changing discourses on institutional dynamics. We briefly position discursive institutionalism (DI) within recent policy analysis literature as our paper fits the DI perspective. The second section depicts the Policy Arrangement Approach (PAA) as an appropriate analytical framework to empirically analyze and understand discursive shifts and institutional dynamics. The third section accounts for our methodological approach. The subsequent section then empirically elucidates the historical analysis of the aforementioned events and their respective impacts on the environmental health discourse and the consequent institutional dynamics. The article concludes with a discussion of the main findings and some suggestions for further research.

### Theoretical background: sensitizing events and discursive institutionalism

As illustrated by Wiering and Arts [3], events that provoke serious societal and political concerns (in their case: a near-flooding disaster in the Netherlands) can cause institutional renewal and shape the ideas and plans for future decision-making and risk management. Discourses are defined by Hajer and Versteeg [4] as, “an ensemble of ideas, concepts and categories through which meaning is given to social and physical phenomena.” Through interaction, agencies exchange discourses, merge into discursive coalitions or split-up into discursive oppositions, depending on shared or conflicting definitions, beliefs, concepts, assumptions, and ideas which can vary over time [5,6]. Discourses gradually solidify into institutional arrangements when successful storylines, advocated by an influent discourse coalition, find their way into policy programmes, measures, practices, budgets, responsibilities, competencies, and rules.

Compared with the other theoretical perspectives within social sciences to study institutions - i.e., Rational Choice Institutionalism, Historical Institutionalism, and Sociological Institutionalism [2] - discursive institutionalism emphasizes: 1) the important role of discourses in influencing actors’ preferences, interests and behaviour [7]; and 2) the role of these discourses in assuring institutional stability, while simultaneously triggering and legitimizing institutional change [2]. Discursive institutionalism tends to pay more attention to (a) the cognitive frames of agencies rather than to their material interests; to (b) a more dynamic, agent-centred approach rather than to static and path-dependent patterns; and to (c) the dynamics of valuation and norm setting rather than with static ones [8,9].

Related to the case study of Flanders, a discursive institutional perspective is taken into account. Our hypothesis is that environmental health events can indeed induce discursive renewal, which in turn, can explain changes in the institutionalization of the Flemish environmental health arrangement.

### Analytical framework: policy arrangement approach

In order to understand why discourses gain dominance while other understandings are discredited and to study the impact of these discourses on institutional dynamics, the Policy Arrangement Approach (PAA) is identified as an appropriate analytical framework. The PAA is developed to empirically analyze change and stability within policy arrangements [5] and tested in a series of research endeavours in the environmental domain.

A policy arrangement is defined by Leroy and Arts [10] as: “The temporary stabilization of the content and organization of a particular policy domain at a certain policy level.” These processes of temporary stabilizations are often referred to as “ongoing processes of institutionalization” [11]. While studying institutional change (or the lack thereof), the PAA distinguishes four interwoven dimensions of any arrangement: (a) the actors and their coalitions involved in the policy domain, (b) the allocated resources and the differences in power that result from their distribution, (c) the formal and informal rules of the game, and (d) discourses. As such, the PAA aims to focus on intentions, motives, discourses, beliefs of actors, formal and informal regulatory roles and organizational structures, but also takes into account long-term processes that characterize contemporary society. Each of these four dimensions might be a trigger of change, while, equally, each of them might block such a change and preserve institutional stability. As mentioned above, this paper focuses on the change-inducing roles of discourses caused by environmental health incidents on the institutionalization of the Flemish environmental health arrangement.

## Methods

It is out of scope to analyse all local Flemish environmental health events that occurred between the 1970s till the 1990s. Four cases were selected, which, according to the interviewees and the results of the document analysis, caused serious environmental health concerns at the societal, the scientific and the political level. These cases are related to: 1) lead pollution from metallurgic activities in Hoboken, 2) cadmium pollution due to zinc smelters in the Northern Kempen, 3) the dioxin deposition by two waste incinerators in Wilrijk, and dioxin contamination in the food chain (4). The first three cases represent human activities with long-term, negative side effects for the environment and human health. The fourth event was a short term, unintended ad hoc contamination of the food chain with potential immediate health effects.

To determine how and to what extent each of these events contributed to discursive changes and to investigate their impact on the Flemish environmental health arrangement, we engaged in a thorough discourse analysis. Data are gathered, analysed, and interpreted according to a qualitative approach, and using a triangulation of methods. Triangulation refers to a well-thought combination of several research methodologies, here document analysis and in-depth interviews, in order to validate the findings through cross verification and to increase the credibility of the results. The document analysis gives some initial indications about historical developments and evolutions, key events, discourses, actors and formal rules of the game. This information is used as an input for the in-depth interviews to ensure well-documented and focused preparations. The interviews, in turn, are necessary to validate the information from the document analysis and to gain additional information as documents tend to only describe the reached compromises and rarely the nuanced information about the discussion behind them, nor the informal rules of the game. As such, both methods are complementary and their combination (in social sciences: triangulation) is necessary in order to increase the credibility and validity of the results.

A valid discourse analysis asks for a clear methodology on how to select documents and interviewees. As to the former, primary and secondary sources are studied depending on the date of incidence occurrence. For the events that occurred before 1985, primary sources are scarcely available or difficult to access. These cases are analysed using secondary sources. Events after 1985 are primarily studied based on original research papers and (doctoral) dissertations. If available, policy documents, legislations, annual reports, and advisory reports are also studied. Additionally, the Flemish newspapers are screened in order to identify important actors, measures, and events but also different stakeholders’ opinions and to verify the historical description of each case. The newspapers are screened using Mediargus, an online press database covering all Flemish newspapers from 1988 onwards, hence not covering the first two events as these date from some decades ago. This paper, however, does not intend to cover the entire history of these events, yet focuses on their impact in terms of changing discourses and institutional dynamics. A detailed overview of the consulted documents is listed in Additional file 1.

**Figure 1:**
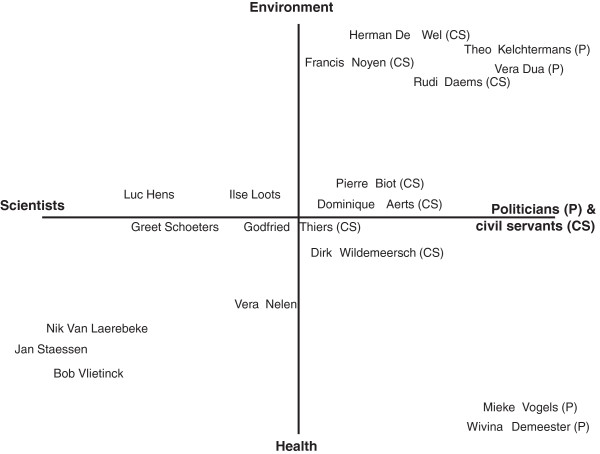
**Graphic overview of the positions and backgrounds of the interviewees.** The interviewees are positioned in a two-axes matrix. The horizontal axis represents the duality between people originating from science or from the political sphere (including civil servants). The vertical axis distinguishes between the domains the interviewees originate from: environment or health. In either case, some respondents restrict to the position we plotted them on, whereas others deliberately cross the boundaries between the domains.

The respondents are selected based on their role and background. Built on two axes, Figure 1 presents an overview of the interviewees, either located in the scientific or the political sphere, and engaged either in the environment or the health domain – with a few of them crossing the boundaries between these fields. Table 1 lists the function of each interviewee. All interviews are semi-structured. The interview guide is based on the results of the document analysis and the outcome of earlier interviews. The questions relate to the agenda setting of environmental health in Flanders, the impact of different events, the role different actors played, the perceived interaction between science and policy, changing discourses about complexity and uncertainty, etc.

**Table 1:** Overview of the functions of the interviewees

**Respondent**	**Function**
Wivina Demeester	Flemish Minister of Health, 1995-1999
Theo Kelchtermans	Flemish Minister of Environment, 1988–1992; 1995-1999
Mieke Vogels	Flemish Minister of Health, 1999-2003
Vera Dua	Flemish Minister of Environment, 1999-2003
Rudi Daems	Principle Private Secretary of the Flemish Minister of Environment, 1999-2003
Pierre Biot	Civil servant, Federal Public Service Health, Food Chain Safety and Environment
Bob Vlietinck	Professor, Genetic Epidemiology
Francis Noyen	Environment and Nature Council of Flanders
Nik Van Larebeke	Professor, Cancer Prevention
Jan Staessen	Professor, Molecular and Cardiovascular Research
Dominique Aerts	Civil servant, Flemish Environmental Administration, late 1990s
Dirk Wildemeersch	Civil servant, Flemish Health Administration
Vera Nelen	Provincial Institute for Hygiene of Antwerp
Greet Schoeters	Flemish Institute of Technological Research – human biomonitoring
Ilse Loots	Professor, Environmental Sociology
Luc Hens	Professor, Human Ecology
Herman De Wel	Civil servant, Flemish Environmental Administration, 1980s-1990s
Godfried Thiers	Manager, Scientific Institute of Public Health, 1980s-1990s

## Results

This section presents for each of the four events, two elements: first, a summary of its history and its stakes; second, an assessment of its impact on the environmental health discourses and the institutionalization of the Flemish environmental health arrangement.

### Lead pollution from metallurgic activities

In 1887, a lead and de-silvering plant opened in Hoboken to refine minerals and extract metals from waste. The complaints of some neighbours in the mid**-**1960s about the air and smell pollution and the large-scale destruction of honeybees could not force general public agitation and political attention. The limited reaction can be explained by the dependency of the population on employment and the priority the Ministry of Labour placed on employment instead of occupational health and safety. In the early 1970s, after the attributed death of six cows and two horses to the lead contamination of the nearby non-ferrous industry, the metallurgical industry was increasingly recognized as not only dangerous for occupationally exposed people, but also for the people living in the neighbourhood [12]. However, there was no scientific proof yet about the possible adverse health effects. Pressured by local action committees and the media, a first small-scale biomonitoring survey was done in 1974 with eleven-year-old children. Based on the same research data, the scientists responsible for this biomonitoring survey set up a contradictory communication strategy. Regarding the local authorities, they communicated there was no reason to panic because the exposure levels were lower than the acceptable limits, while at the same time, they concluded in a peer-reviewed scientific journal [13] that children were a more vulnerable group than adults and that the average measured concentration of lead in children’s blood (30.1 μg/dl) was higher than the maximum biologically allowable concentration (25 μg/dl). Consequently, the local authority established a local medical experts’ group to review and reanalyse the earlier published studies. Based on its recommendations, a general biomonitoring screening of the local population every two years was set up. The first biomonitoring research results were much worse than expected: the average lead concentration in blood (34 μg/dl) greatly exceeded the maximum biologically allowable concentration. Furthermore, the assumption was made that long-term lead exposure could cause negative cognitive effects, such as mental retardation. After the formal confirmation of the lead pollution in Hoboken and the revelation in the media, the Ministry of Public Health developed an action programme in 1978 in order to clean-up the contamination area. As part of the action programme, biannual biomonitoring surveys of school-age children were set up and a ministerial working group was established composed of experts and representatives of the relevant ministries. The lead concentration in blood decreased gradually from an average of 40 μg/dl in 1978 to 24.3 μg/dl in 1984. It is difficult, however, to determine whether this reduction is the result of a good working action plan or of changing industrial production processes.

What lessons has one learned from the historical analysis of the lead case? The lead pollution in Hoboken is a typical example of a long-existing problem which became a risk event after the suspicious death of cattle, supported by a chemical analysis of the lead content in the deceased animals’ organs, the hay feed, and the soil [12]. Although these research results did not confirm a negative impact on public health, it was an early warning, to say at least. In addition, this revelation dates from the early 1970s, the era of rapidly growing environmental concern worldwide. As to the environmental health discourse, two shifts are identified. First, through the years, the complexity of the problem, the potential long-term effects on health and well-being caused by the accumulation of lead in the human body and the related scientific uncertainty had increasingly been recognized. As a consequence, there is a slowly growing appeal for a more integrated approach. Second, one had been realizing that a norm based on the average cannot protect all humans equally, as school-age children were assessed as a more vulnerable group. These discursive shifts resulted in some preliminary actions in (1) the knowledge-production process (i.e., state-owned research institutions on public health were authorized to scientifically investigate the health impact of lead contamination and set up small-scale follow-up biomonitoring research of school-age children every two years), (2) as well as in the environmental health policy arrangement (i.e., the establishment of an inter-ministerial working group at the Flemish governmental level as a first step toward policy coordination), and (3) the science-policy interface (i.e., the set-up of a medical experts’ group to advice local governments and industry based on scientific evidence).

### Cadmium pollution due to zinc smelters

In the mid-twentieth century, Belgium was an important producer of zinc. The zinc smelters in the Northern Kempen and Liège had been emitting cadmium, as a by-product, into the atmosphere since 1888. In the 1970s, new technologies were used, resulting in a decrease of the cadmium emission. However, as a consequence of the estimated elimination kinetics of cadmium in humans, residents in the affected areas were still suffering the historic cadmium pollution [14].

At the end of the 1970s, three scientific studies were performed in the Liège area in order to assess whether cadmium pollution in the environment results in an increased uptake of cadmium in the human bodies and the development of health effects. Until that time, only animal experiments were done. These three studies determined higher levels of cadmium in blood and urine in the contaminated area [15], a higher mortality rate for renal diseases possibly influenced by environmental factors [16], and higher accumulated cadmium levels in the renal cortex and liver, notwithstanding differences in occupational exposure or smoking habits [17]. Nevertheless, the scientists communicated their results very carefully as all studies have been performed in the same area and there might be an unknown interfering factor [14]. As a consequence, a large-scale, cross-sectional epidemiological study was launched in de mid-1980s. The main conclusions were that environmental exposure to cadmium may induce renal tubular dysfunction but it could not be confirmed that increased cadmium exposure is related to blood pressure elevation and a higher prevalence of cardiovascular diseases [18]. Although the political impact of the study was limited, the study was at least scientifically innovating as a large-scale human biomonitoring screening was performed in a relatively wide region to quantify exposure of the general population to an environmental contaminant. The cooperation between epidemiologists and biologists was considered as an added value in order to better assess the potential health impact of environmental pollutants [14].

A follow-up research was undertaken in the early 1990s in order to investigate how exposure changes over time. Additional measurements were done related to bone metabolism and calcium homeostasis. The main finding stated that even at a low degree of environmental exposure, cadmium may promote skeletal demineralization [19]. The local government was afraid that the land value would decrease and therefore minimised the results. Anticipating the clean-up operation of the polluted soil in the Northern Kempen, the Flemish Government established two sensitization campaigns in 1994 and 1999 advising the inhabitants to use tap water, to apply hand hygiene, and to refrain from eating locally grown vegetables.

What lessons has one learned from the historical analysis of the cadmium case? Similar to the lead case, the cadmium pollution is also characterized by a long-term problem which evolved to a risk event in response to an increase of environmental concern worldwide. As to the environmental health discourse, this event strengthened the idea that also long-term, low-dose exposure may affect human health. As to the knowledge-production process, more large scale human biomonitoring surveys were set up investigating the general population (and not only school-age children), in different areas that were environmentally (and not occupationally) exposed to cadmium. Moreover, people were followed over the years in order to investigate how exposure changes over time and to determine possible long-term health effects. Additionally, the cadmium case made it clear that epidemiologists, toxicologists and biologists have to cooperate in order to better assess the potential health impact of environmental pollutants. Although the lead case already made it clear that ministerial cooperation is necessary to solve environmental health problems, there is no indication of concerted action found in the cadmium case. It seems as if the fragmentation of competencies between different governmental levels and a tradition of strict demarcation of ministers’ competencies in Belgium still hampered policy coordination.

### Dioxin deposition by two waste incinerators

Since the 1970s, more and more municipal waste was incinerated causing air pollution. During the early years of incineration, these emissions were mostly not purified [20]. Despite the smell and dust nuisances, the general public was less concerned as long as the smokestacks were high enough. In contrast to the lead pollution in Hoboken, neighbours of incinerators apparently did not fear a land value decrease due to incineration. In 1997, however, neighbours of the Wilrijk (Antwerp region) waste incinerator suddenly became concerned about the toxic emissions of two municipal waste incinerators in their residential area. Strengthened by scientific journalists’ information, they started to link cancer, congenital malformations and genetic anomalies to the emission of dioxins by these incinerators. Initially, those complaints were brushed aside by the local mayor, deferring to the lack of scientific certainty. A scientific difficulty was that the region was surrounded by busy high ways, by non-ferrous industry and some other sources of environmental pollution [21]. Under this societal pressure, the local government of Antwerp set up a health survey in the residential area in 1995. The research team identified eight genetic anomalies, but was not able to determine whether these malformations were a coincidence or could be attributed to the incinerators. The local inhabitants, local physicians, local environmental action groups, and even scientists criticized the survey and expressed different, often conflicting opinions in the media. Consequently, the then Flemish Minister of Public Health financed two public health studies to determine whether there was a causal relationship between those health effects and dioxin pollution. The results of both studies [22,23] were questioned by other scientists and protest committees because of the many uncertainties, the lack of statistical significance, the methodology used, and the way the results were communicated and information was suppressed to prevent commotion. Instead of an objective, univocal scientific advice, scientific controversy ensued. In the meanwhile, and in order to temper public concern, the then Flemish Minister of the Environment temporary closed down the incinerators which were exceeding the dioxin emission limit, a decision referring to the Precautionary Principle. An incinerator could only restart its activities after the unanimous permission of the Baeyens’ Committee, an independent scientific advisory board consisting of engineers and medical experts [21]. However, the members of the committee had different opinions about whether to close or start up the incinerators again.

What lessons has one learned from this case study? As to the environmental health discourse, one recognized novel threats, i.e., the unborn child and the fertility. One also learned that a technical approach – for instance, to raise the smokestack – was not an adequate solution to this type of problems. Scientific controversy and uncertainty hampered the political and social debate. Since the shortcomings of different, ad hoc, short-term disciplinary studies were recognized, one expressed the need for a systematic, integrated assessment approach by a multidisciplinary research team of environmental, technical and medical experts. Next to these, social and communication scientists could help improving the communication strategies. In brief: one recognized the need for a better coordination of environmental and public health policies, acknowledged the added value of policy-oriented, interdisciplinary research (teams), and the valuable contribution of social and communication scientists. These lessons were a clear stimulus to take some first steps toward the institutionalization of the Flemish environmental health policy arrangement: the establishment of complaint desks and the organization of scientific policy support. Both were important measures to trace local worries as soon as possible and to manage them efficiently.

### Dioxin contamination in the food chain

In January 1999, a mixture of polychlorinated biphenyls (PCBs) and dioxins was unintentionally mixed with recycled fats used for animal feeds in poultry, swine, and cattle farms in Belgium resulting in a drop in egg production, a reduction in egg hatchability, and an increased mortality of chicks [24,25]. The scientific confirmation of the cause of the dioxin contamination, however, was not forthcoming until the end of April 1999 [25] as the trace within the food chain was very difficult due to various illegal, and black market practices [26]. In May 1999, just a few weeks before the general elections, the contamination was leaked to the media [27,28]. The large latency period between the first signs of the problem and informing the public, the scientific controversy about the possible health consequences, and the uncertainty about the number of exposed individuals [26], triggered a major political and food crisis. The media widely echoed complaints from the society that the Belgian Government was serving the interests of farmers’ unions and the meat industry. In addition, politicians were accused of trying to protect their own interests, anticipating the general elections in June, instead of protecting public health [29,30]. In fact, and even though no actual health effect could be determined, the events turned into a major political crisis and forced the government to drastic measures. The Federal Ministers of Health and Agriculture were forced to resign; a massive international recall operation of eggs and chicken, followed by almost all meat products took place [24], the slaughter and transport of poultry, cattle, and swine were prohibited [26]; an embargo was placed on all Belgian food products of animal origin; and tons of eggs and meat products were destroyed [30]. Although there was no single health impact, the economic damage was enormous and the political costs in terms of distrust were, if possible, even higher.

The dioxin contamination in the food chain strongly influenced the elections of June 1999. The governmental parties (the centre-left Christian-Democrat/Socialist coalition) lived an electoral defeat, while the opposition parties (the Liberals and the Green Party) pulled votes. The Green Party was associated with food safety, healthy and biological food and would join the newly formed government 1999–2004.

Responding to the excessive public concern, the new Federal Government (a) appointed a crisis manager to coordinate governmental action, (b) established an independent scientific committee, (c) integrated the Belgian inspection services into one agency responsible for the whole food chain, and (d) launched a systematic monitoring programme for food of animal origin [26]. Together, these measures should detect possible food problems immediately, manage them efficiently, and communicate appropriately about them [24].

What lessons has one learned from this case study? Contrary to the three previous cases that dealt with rapidly upcoming concerns about a longer existing exposure to environmental pollution, the dioxin contamination in the food chain can be considered as a short-term, yet almost instantly all-pervading concern. The latter was fuelled in part by hush-hush policy response itself. Moreover, the dioxin incident demonstrated that risk perception among the general public is not primarily driven by scientific evidence, yet strongly influenced by the media and socio-psychological factors. In line with the previous cases, the dioxin contamination again emphasized the complexity of environmental health problems and the need for integrated knowledge-production and policy-making processes. And as the previous events it appealed for an organizational set up that could detect problems and/or concerns as early as possible and that could develop effective surveillance systems and communication strategies.

## Discussion

The central aim of this paper is to assess the extent to which four local events that triggered serious environmental health concerns have contributed to discursive changes, which in turn, affected the institutionalization process of the Flemish environmental health arrangement. To this end, the discursive changes are firstly summarized and followed by their impact on: 1) the scientific organization and methodologies for knowledge production; 2) the relationship between science and policy; and 3) the risk communication and risk management strategies, including policy coordination between the environmental and public health policy fields. Finally, we reflect upon the theoretical and analytical concepts and the empirical research scope. This section also suggests some future research topics.

### Changing discourses on environmental health risks

Each of the four events reported on has demonstrated scientific knowledge gaps and scientific controversy about the impact of environmental pollution on public health. A first explanation refers to the novelty of the environmental health discipline. Second, also the complexity of environmental health problems increases scientific uncertainty and ambiguity. After all, these problems are characterized by multiple sources, multiple agents, multiple pathways, multiple exposure routes, multiple health effects, long delay periods between cause and effect, cross-bordering scale effects, etc. A third contribution is linked with the interwoven character of environmental health problems in a larger context of economic, financial, and social values resulting in a variety of divergent problem definitions and risk perception. For instance, in the lead case the inhabitants almost denied health problems being afraid of losing their jobs and decreasing the value of their houses, while, in contrast, the people living in the neighbourhood of the waste incinerators almost claimed health damage caused by the incineration activities. Moreover, the environmental health discourse gradually shifted from mortality and severe health effects caused by a short-term, high-dose exposure to moderated health effects, negative effects on well-being, the unborn child and fertility in response to a long-term (low-dose) exposure (e.g., cognitive effects in the case of lead exposure, osteoporosis as a result of cadmium exposure, and congenital anomalies in children living around the waste incinerator). Through the four cases, a new discourse was shaped emphasizing the need to differentiate various target groups (school-age children, foetuses and breastfed babies) and to differentiate environmental quality standards as the average cannot protect all humans equally.

### Institutional challenges related to scientific knowledge production

The discursive shift in response to scientific uncertainty and controversy has influenced the institutional context of the knowledge-production process throughout the four cases. Moreover, the scope of environmental health research has extended from one pollutant to complex mixtures of pollutants and from the focus on one part of the chain to the whole chain of causes and effects. As a consequence, the knowledge-production process requires a more integrated approach at the organizational (interdisciplinary research teams) as well as methodological (integrated risk assessment) level. For instance, this series of events gradually illustrates the need for continuously broadening the number of scientific disciplines which should be included in interdisciplinary research teams: from cooperation between toxicologists and epidemiologists to the appointment of biologists, statisticians and engineers. Moreover, social scientists and communication experts were considered necessary to better understand risk perception and the social processes and social responses related to this. This, in turn, could help to set up more appropriate participatory and integrative processes as well as to improve communication processes. It must be noted, however, that the discussion about integrated knowledge production is limited to a range of scientific disciplines. The need to include other types of expertise and knowledge, e.g. lay-knowledge as defined by Wynne [31], was not yet explicitly recognized at that time. At the methodological level, the four cases gradually demonstrate the increased recognition for integrated environmental health impact assessment methods [32], taking into account the entire cause-effect chain of environmental health problems. Biomonitoring is recognized as a complementary, feasible research method next to epidemiology, toxicology, and the measurement of pollutants in the environment. Throughout the four cases, biomonitoring was used for an increasing number of pollutants, following-up the same people across different time periods and a wider geographical research area. As a self-evident part of these novel methodological developments, research projects have been strongly driven by discussions about uncertainty. As such, the debate about uncertainties has evolved from an epistemological aspect, to a methodological question about how to scientifically deal with uncertainties. To summarize, the epistemology and methodology on environmental health problems have more characteristics of the Post-Normal Science epistemology, in which the quality of the knowledge production process is as much important as the validity and reliability of the knowledge itself [33].

### Science-policy interface

The changing epistemological discourses have also influenced the science-policy interface. Initially, the Government established (technical) expert committees to depoliticize the environmental health problems and to legitimize political decisions. However, the complexity of the problems challenged the Government to develop new strategies to deal with scientific uncertainty and to legitimize the political decisions as scientists were not able to produce univocal conclusions in the short term. During the lead and cadmium crisis, the Belgian Government (mis)used scientific uncertainty to delay the decision-making process and pass over the problem in order to prevent panic. The establishments of multi-disciplinary working groups, in which representatives from different ministries and administrations, experts, local authorities, professionals and even local and national environmental groups were invited to participate, illustrate that it was not sufficient anymore to only involve politicians and scientists to deal with this type of complex problems. During the incinerator case, the Precautionary Principle was applied in response to the challenges of scientific uncertainty. The dioxin crisis in the food chain, at last, indicated that the application of the Precautionary Principle in the case of complex problems is insufficient. After all, one can take drastic measures and shut down a waste incinerator, but for a food chain it is (a) far more difficult to detect the cause of contamination in such a complex chain, and (b) far more difficult to intervene as that should encroach deeply on social life. Referring to Hoppe [34], first indications towards a Model of Mutual Learning are identified. A broader interpretation of stakeholder participation has been emphasized to legitimize the knowledge-production as well as the decision-making processes.

### Communication and management of environmental health risks

The analysis of these events demonstrates how the debate about uncertainty has evolved from an epistemological aspect, to a methodological question about how to scientifically deal with uncertainty, and finally to a challenge for governments about how to manage and communicate uncertain environmental health risks.

Related to risk communication, all cases were characterized by a lack of transparency by the governmental authorities, although, the importance of communication was already emphasized during the lead case in the 1970s. Through the four cases, medical, environmental and technical scientists as well as politicians became more aware of the importance of consulting social and communication scientists and to organize local information meetings in order to present scientific information to the general public, explain them the significance and meaning of the information, and listen to the concerns of the stakeholders.

In order to effectively manage environmental health problems, the quick succession of incidents illustrates an increased need for: 1) the establishment of an organizational structure in order to detect environmental health problems as fast as possible, and 2) policy coordination and integration between the environmental policy and health policy fields (also called multi-sector governance). Related to the early detection of environmental health concerns, the first complaint desk was established after the incinerator incident, bridging the gap between the general public and the Flemish authorities. In order to manage environmental health problems effectively and efficiently, the results of the case study analysis emphasize the need to better coordinate and integrate the efforts done by the different administrations within the Flemish Ministry, more precisely between the Administration of Public Health and the Administration of the Environment by establishing ministerial working groups.

### Research scope

The aim of this research paper was to focus on the impact of local events that triggered environmental health concern on discursive dynamics and the institutionalization of the Flemish environmental health arrangement. Consequently, neither the mainstream developments in the international literature nor comparable local events in neighbouring countries belong to its scope. This does not imply that we regard these as irrelevant: European Directives (e.g., on particulate matter) have a clear impact on Flemish environmental legislation. However, we believe that these four local events have been necessary to set environmental health on the Flemish political, scientific and societal agenda. It seems as if, through these events, Flanders went through the phases in risk thinking that one reports on in international literature. The National Research Council, for instance, distinguished five steps: (1) getting the science right, (2) getting the right science, (3) getting the right participation, (4) getting the participation right, and (5) developing accurate, balanced, and informative synthesis [35]. Although not planned in advance, this comes very close to what happened in Flanders as the results of the series of environmental health events.

### Analytical and methodological reflection

The combination of a discursive institutionalism stance and a historical analysis based upon the Policy Arrangement Approach enabled to study how environmental health problems have shaped thinking, research and policies, leading onto the institutionalization of the environmental health arrangement. The role and impact of this type of events on institutional dynamics is underestimated in the scholarly literature. This paper gives an empirical contribution to this discussion and is important for understanding and improving risk governance. Each analysed event shows evidence of some sort of learning and knowledge development because it opens the eyes and minds, while the accumulation of these events within a short time period, shortly followed by elections, was necessary to achieve institutional change. The fact that these incidents occurred within a short time period is of crucial importance, as the attention of the general public, media and policymakers is likely to be quite short-lived.

For this type of research, the Policy Arrangement Approach turned out to be a helpful analytical framework. First, the PAA allows the understanding and explanation of the development, change and continuity of a policy arrangement. Second, the approach examines the content as well as the organizational structure, which both influence institutional stability or change. An alternative analytical framework would have been the framework developed by Runhaar et al. [36,37] for characterizing, explaining, and evaluating environmental health risk governance regimes. The latter emphasizes the judicial approach, primarily focusing on the rules of the game and on procedures. As such, the impact of discourses is given less attention, while Hajer [7] and Scott [2] already elucidated the pivotal role of changing discourses in explaining institutional dynamics.

The combination of content analysis of documents and in-depth interviews with the main stakeholders resulted in a detailed and balanced understanding of the impact of the series of environmental health events on discursive change and institutional dynamics. The strength of this data triangulation approach is its internal validity. Contrary, the external validity of the research results is rather limited. The results cannot be generalized either to other regions and countries, or to other policy sectors. Comparative historical analyses would be interesting for further research in order to provide a scientific contribution to the theoretical discussion on the role and impact of events on institutional dynamics. Moreover, a cross-regional/national analysis would increase mutual learning and provide a more detailed study on the extent to which emerging (inter)national discourses leads to changes in institutional arrangements of other countries or regions.

## Conclusions

This study shows how environmental health problems have shaped thinking, research, policies and the institutionalization of the Flemish environmental health arrangement. The empirical analysis indicates that a series of events resulted in a gradual discursive shift, which in turn, affected the institutionalization of the Flemish environmental health arrangement. The major institutional challenge was to increase integrated environmental health knowledge production and decision making in order to systematically deal with complex and uncertain environmental health problems. Integration at the epistemological and methodological level refers to (i) knowledge production in interdisciplinary research teams; (ii) the involvement of social scientists and communication experts to better understand the social aspects related to environment and health problems and, from there, to improve participatory processes and communication strategies; (iii) appropriate problem framing by all stakeholders; (iv) the usage of integrated environmental health impact assessment tools, taking into account serious environmental health effects as well as negative effects on well-being, and differentiating between vulnerable groups; and (v) appropriate uncertainty management. All these characteristics remind one of the Post-Normal Science epistemology of Funtowicz and Ravetz [33] and the ideas of integrated environmental health impact assessment as proposed by Briggs [32]. Integration at the governmental level refers to the participation of stakeholders and the integration of different types of knowledge (and not only expert-knowledge) in the decision-making process, on the one hand, and the integration of environmental health objectives in all relevant policy domains, on the other. The analysis also emphasized the need to develop an effective communication strategy in interaction with social experts and the establishment of an organizational structure to detect environmental health problems as fast as possible and to react immediately, in order to prevent the evolvement of environmental health events into crisis. As a consequence of the epistemological and governmental discursive and institutional shifts, the consecutive events also indicate changes in the science-policy interface towards mutual learning in which all relevant stakeholders are involved. To conclude, the quick succession of risk events sowed the seeds for the institutionalization of a Flemish environmental health arrangement, translating new discourses into organizational structures, new resources, legislation, rules of the game, etc. The historical analysis is important for understanding and improving risk governance arrangements nowadays.

## Abbreviations

DI: Discursive institutionalism; PAA: Policy arrangement approach; PCB: Polychlorinated biphenyls.

## Competing interests

The authors declare that they have no competing interests.

## Authors’ contributions

KRS was responsible for the empirical data gathering (document analysis, in-depth interviews) and the interpretation of data. KRS and PL have made substantial contributions to conceptions, design and methodology. RS have been involved in revising the draft paper critically. All authors read and approved the final manuscript.

## Supplementary Material

Additional file 1 List of consulted documents. Click here for file
