# Supplementary material for: Sensitizing events as trigger for discursive renewal and institutional change in Flanders’ environmental health approach, 1970s-1990s
Source: Environ Health. 2013 Jun 7;12:46. doi: 10.1186/1476-069X-12-46 (PMC3681675; doi:10.1186/1476-069X-12-46)
Supplement: Additional file 1 — List of consulted documents. [file 1476-069X-12-46-S1.pdf]

- **Research reports and scientific articles**

Aelvoet A, Nelen V, Schoeters G, Vanoverloop J, Wallijn E, Vlietinck R: **Risico op gezondheidsschade bij kinderen in de Neerlandwijk te Wilrijk.** [VITO report 1998/TOX/R/030]. Mol: VITO; 1998.

Bernard A, Broeckaert F, De Poorter G, De Cock A, Hermans C, Saegerman C, Houins G: **The Belgian PCB/Dioxin Incident: Analysis of the Food Chain Contamination and Health Risk Evaluation.** *Environ Res2002,* **88***:* 1-18.

Bernard A, Fierens S: The Belgian PCB/Dioxin Incident: A Critical Review of Health Risks Evaluations. *Int J Toxicol* 2002*,* 21: 333-340.

Blootstellingsonderzoek Noorderkempen. Geïntegreerd rapport. Brussel: Vlaamse overheid; 2008.

Buchet JP, Lauwerys R, Roels H, Bernard A, Bruaux P: **Renal effects of cadmium body burden of the general population**. *Lancet* 1990*,* **336**:699-702.

Crabbé A: De rol van ministeriële kabinetten in het politieke besluitvormingsproces [Msc dissertation]. Antwerpen: Universiteit Antwerpen; 2000.

Craye M, Goorden L, Van Gelder S, Vandenabeele J: **Milieu en Gezondheid: naar een adequate dialoog tussen overheid, bevolking en wetenschap.** Antwerpen: Universiteit Antwerpen; 2001.

Diricks H: **The dioxin crisis in Belgium: from crisis to scientific knowledge and a new policy.** Presentation at the International Public Health Symposium on Environment and Health Research, WHO, Madrid, 20-22 October; 2008.

Jans HWA, Van den Hazel P: **Medische milieukunde in Vlaanderen**; 1999.

Keune H, Craye M: **Schijnbare paradoxen over milieu en gezondheid**. *Kwartaaltijdschrift Economie* 2004*,* **1**:225-244.

Keune H, Mertens R, Goorden L, Loots I: **Onrust in Moretusburg? Risicocommunicatie met de bevolking naast de fabriek.** Antwerpen: Universiteit Antwerpen, Steunpunt Milieu & Gezondheid; 2002.

Lauwerys R, Amery A, Bernard A, Bruaux P, Buchet JP, Claeys F, De Plaen P, Ducoffre G, Fagard R, Lijnen P, Nick L, Roels H, Rondia D, Saint-Remy A, Sartor F, Staessen J: **Health Effects on Environmental Exposure to Cadmium: Objectives, Design and Organization of the Cadmibel Study: A Cross-Sectional Morbidity Study Carried Out in Belgium from 1985 to 1989**. *Environ Health Perspect* 1990, **87:**283-289.

Lauwerys R, Bernard A, Buchet JP, Roels H, Bruaux P, Claeys F, Ducoffre G, De Plaen P, Staessen J, Amery A, Fagard R, Lijnen P, Thijs L, Rondia D, Sartor F, Saint-Remy A, Nick L: **Does environmental exposure to cadmium represent a health risk? Conclusions from the Cadmibel study**. *Acta Clinica Belgic*a 1991, **46**:219-225.

Lauwerys R, De Wals P: **Environmental pollution by cadmium and mortality from renal diseases**. *Lancet* 1981, **317**:383.

Lauwerys R, Hardy R, Job M, Buchet JP, Roels H, Bruaux P, Rondia D: **Environmental pollution by cadmium and cadmium body burden: an autopsy study**. *Toxicol Lett* 1984, **23**:287-289.

Léonard A, Deknudt GH, Debackere M: **Cytogenetic investigations on leukocytes of cattle intoxicated with heavy metals**. *Toxicology* 1974*,* **2***:*269-273.

Leroy P: **Herrie om de heimat: milieuproblemen, ruimtelijke organisatie en milieubeleid** [Doctoral Thesis]. Antwerpen: Universiteit Antwerpen; 1983.

Loots I, van den Broek J, Leroy P: **Vlaams milieubeleid na 1980: een schets van de institutionalisering**. In *Milieubeleid: theorie en praktijk*. Edited by Cörvers R. Heerlen: Open Universiteit Nederland, 2009:15-34.

Nawrot T, Plusquin M, Hogervorst J, Roels HA, Celis H, Thijs L, Vangronsveld J, Van Hecke E, Staessen JA: **Environmental exposure to Cadmium and risk of cancer: a prospective population-based study**. *Lancet Oncol* 2006*,* **7**:119-126.

Nawrot T, Van Hecke E, Thijs L, Richart T, Kuznetsova T, Jin Y, Vangronsveld J, Roels HA, Staessen JA: **Cadmium-Related Mortality and Long-Term Secular Trends in the Cadmium Body Burden of an Environmentally Exposed Population.** *Environ Health Perspect* 2008*,* **116**:1620-1628.

Nemery B, Fischler B, Boogaerts M, Lison D, Willems J: **The Coca-Cola incident in Belgium, June 1999**. *Food Chem Toxicol* 2002, **40**:1657-1667.

Nouwen J, Cornelis C, De Fré R, Wevers M, Viaene P, Mensink C, Patyn J, Verschaeve L, Hooghe R, Maes A, Collier M, Schoeters G, Van Cleuvenbergen R, Geuzens P: **Health Risk Assessment of Dioxin Emissions from Municipal Waste Incinerators: the Neerlandquarter (Wilrijk, Belgium)**. *Chemosphere* 2001, **43**:909-923.

Onderzoek naar factoren die loodbloedgehalten van kinderen in Moretusburg beïnvloeden. Wilrijk: Universiteit Antwerpen; 2002

PIH: **Verslag betreffende het onderzoek op zware metalen in de omgeving van de soc. Gen. Met. De Hoboken S.A.** Antwerpen: Provinciaal Instituut voor Hygiëne; 1973.

Roels H, Bruaux P, Buchet JP, Claeys-Thoreau F, Lauwerys R, Lafontaine A, Hubermont G, Van Overschelde J: **Impact of air pollution by lead on the heme biosynthetic pathway in school-age children**. *Arch Environ Health* 1976, **31**:310-316.

Roels HA, Lauwerys R, Buchet JP, Bernard A: **Environmental exposure to cadmium and renal function in aged women in three areas of Belgium**. *Environ Res* 1981, **24**:117-130.

Staessen JA, Amery A, Bernard A, Bruaux P, Buchet JP, Bulpitt DJ, Claeys F, De Plaen P, Ducoffre G, Fagard R: **Blood Pressure, the Prevalence of Cardiovascular Diseases, and Exposure to Cadmium: a Population Study**. *Am J Epidemiol* 1991*,* **134**:257-267.

Staessen JA, Buchet JP, Ginucchio G, Lauwerys RR, Lijnen P, Roels H, Fagard R: **Public health implications of environmental exposure to cadmium and lead: an overview of epidemiological studies in Belgium**. *J Cardiovasc Risk* 1996*,* **3**:26-41.

Staessen JA, Roels HA, Emelianov D, Kuznetsova T, Thijs L, Vangronsveld J, Fagard R: **Environmental exposure to cadmium, forearm bone density, and risk of fractures: prospective population study**. *Lancet* 1999, **353**:1140-1144.

Van Larebeke N: **Health Effects of a household Waste Incinerator near Wilrijk, Belgium**. In *Health Impacts of Waste Management Policies*. Edited by Nicolopoulou-Stamati P, Hens L, Howard CV. Dordrecht: Kluwer, 2000:239-250.

Van Larebeke N, Hens L, Schepens P, Covaci A, Baeyens J, Everaert KJL, Bernheim R, Vlietinck R, De Poorter G: **The Belgian PCB and dioxin incident of January – June 1999: Exposure data and potential impact on health**. *Environ Health Perspect* 2001*,* **109**:265-273.

Verschaeve L, Schoeters G: **Cytogenetisch populatieonderzoek: commentaren bij het cytogenetisch onderzoek van kinderen in de Neerlandwijk** [VITO report 1998/R/TOX/045]. Mol: VITO; 1998.

Vlietinck R, Schoeters G, Van Loon H, Loots I: **Eindrapport van het onderzoek Milieu & Gezondheid. Ontwikkeling van een concept voor de opvolging en risico-evaluatie van blootstelling aan leefmilieupolluenten en hun effecten op de volksgezondheid in Vlaanderen**; 2000.

- **Policy Letters, Policy Plans, Policy Documents**

Environment and Nature Report Flanders (MIRA): 1994, 1996, 1998, 1999, 2000, 2002, 2003, 2004.

Environmental Policy Plan 2003-2007 (MINA 3).

Five-year Policy Letter on Environment and Nature: 1995-1999; 1999-2004; 2004-2009.

Five-year Policy Letter on Welfare, Public Health, and Family: 1999-2004; 2004-2009.

Integrated Policy Letter on Environment and Health, 2000.

Year Policy Letter on the Environment: 1992, 1994, 1997, 1998-1999, 2000-2001, 2001-2002, 2002-2003, 2003-2004.

Year Policy Letter on Welfare, Public Health, and Family: 1995-1996; 1996-1997; 1997-1998; 2000-2001; 2001-2002; 2002-2003; 2003-2004.

Annual publications of the Flemish Ministry for the Environment (LIN – LNE) from 1995 until 2004.

Annual reports of the Flemish Health Council from 2001 until 2004.

- **Legislation and Agreements Concerning Management**

Decreet tot instelling van een Milieu- en Natuurraad van Vlaanderen en tot vaststelling van de algemene regelen inzake de erkenning en de subsidiëring van de milieu- en natuurverenigingen van 29 april 1991.

Decreet van 5 april 1995 houdende algemene bepalingen inzake milieubeleid.

Decreet van 20 december 1996 houdende oprichting van een Vlaamse Gezondheidsraad en van een Vlaamse Adviesraad inzake erkenning van verzorgingsvoorzieningen (BS 15/11/1997).

Besluit van de Vlaamse Regering van 28 januari 1997 tot uitvoering van het decreet van 20 december 1996 houdende oprichting van een Vlaamse Gezondheidsraad en van een Vlaamse Adviesraad voor erkenning van verzorgingsvoorzieningen (BS 4/4/1997).

Decreet van 18 juli 2003 tot de regeling van strategische adviesraden.

Decreet van 30 april 2004 ter aanvulling van het decreet van 5 april 1995 houdende algemene bepalingen inzake milieubeleid met een titel “strategische adviesraad” en tot wijziging van diverse andere decreten.

Flemish Decree on preventive health policy, 2003.

- **Flemish Parliament**

Vlaamse Raad (1984). Bulletin van vragen en antwoorden. Zitting 1983-1984, nr. 7, 31 januari 1984.

Vlaams Parlement (1997). Commissie voor Welzijn, Gezondheid en Gezin, Verslag, Vergadering 23 oktober 1997.

Vlaams Parlement. Maatschappelijke beleidsnota milieu en gezondheid. Parlementaire Ad Hoc Commissie Milieu en Gezondheid, 6 juni 2001. Stuk 740 (2000-2001) – Nr. 1.

Vlaams Parlement. Maatschappelijke beleidsnota milieu en gezondheid. Motie van aanbeveling, 4 juli 2001. Stuk 740 (2000-2001) – Nr. 4.

Flemish Parliament 1994-2009: all documents related to the Commission for the Environment and the Commission for Public Health.

- **Advices from the official advisory boards**

Advies van de Vlaamse Gezondheidsraad dd. 24 maart 1998: advies inzake onderzoeksinspanning en kenniscentrum mbt gezondheidseffecten van milieufactoren.

Advies van de Vlaamse Gezondheidsraad dd. 17 oktober 2000: Prioriteitstelling wetenschappelijk onderzoek op het terrein Gezondheid en Milieu – op vraag van minister Vogels (19.07.2000), Vlaams minister van Welzijn, Gezondheid en Gelijke Kansen.

Advies van de Vlaamse Gezondheidsraad, bevraging eerste nationaal actieplan voor milieu en gezondheid, maart 2003.

Briefadvies van de Mina-Raad dd; 01 februari 2001 over de prioriteiten voor wetenschappelijk onderzoek op het terrein van gezondheid en milieu.

Advies van de Mina-raad dd. 5 april 2001 over de band tussen milieu en gezondheid.

Briefadvies van de Mina-raad dd; 3 april 2003 over het voorontwerp van het Nationaal Actieplan Milieu- en Gezondheid en de Samenwerkingsovereenkomst Milieu en Gezondheid.

Advies SERV over de band tussen milieu en gezondheid, 18 april 2001.

- **Secondary sources and newspaper articles**

d’Aubioul P: **Moretusburg, groene wijk onder de schouwen van de metallurgie.** *De Biomonitor* 2008.

BBL: **Vier milieufederaties (1971-2001): een verhaal van dertig jaar eenheid in verscheidenheid…** BBL: Brussel; 2001

Belga. (25 augustus 1994). **Arsenicumvervuiling: Detiège gaat de bevolking voorlichten.** *Het Belang van Limburg*, p. 7.

Belga (16 januari 1999). **Minister Kelchtermans ontkent verdeeldheid in commissie Baeyens**. *Gazet van Antwerpen*, p. 2.

Bruyneel E: **De Hoge Gezondheidsraad (1949-2009). Schakel tussen wetenschap en volksgezondheid.** Leuven: Uitgeverij Peeters; 2009.

Buyst E, Lowyck K, Soete A: **Al 20 jaar voor het milieu van morgen. Kroniek van de Vlaamse Milieumaatschappij 1991-2011.** Aalst: Vlaamse Milieumaatschappij; 2011.

Ceustermans C. (2 Februari 1998). **Onenigheid over toekomst van Wilrijkse verbrandingsoven**. *De Morgen*, p.8.

Claes P, Hendrikx T, Rutten C, Depauw G, Miciels G: **Honderd jaar zorg voor milieu, natuur en gezondheid in de provincie Antwerpen, 1896-1996. PIH 100 jaar.** Antwerpen: Provincie Antwerpen; 1997.

Cremers P, Van Laerhoven B, Willems R: **Moorddadig milieu in Vlaanderen. Moeder waarom leven wij?** Antwerpen: Standaard Uitgeverij; 1997.

De Swaan A: **Zorg en de Staat. Welzijn, onderwijs en gezondheidszorg in Europa en de Verenigde Staten in de nieuwe tijd**. Amsterdam: Uitgeverij Bert Bakker; 1989.

Donckler E. (31 oktober 1994). **Arsenicumvervuiling in Lommel voorbij?** *Het Belang van Limburg*, p. 1.

Gijsels H: **Als het lood om je hoofd is verdwenen…** Antwerpen: EPO; 1979.

Hendrix J, Reynders G. (24 maart 1999). **Cadmiumgehalte met 30% gedaald.** *Het belang van Limburg*, pp. 1.

Houben F: **De Medisch Milieukundigen...onder de loep genomen.** *Biomonitor* 2004, **2**.

Houben F, Van Peer L: **De Medisch Milieukundigen...de voorgeschiedenis**. *Biomonitor* 2004, **2**.

Kempische bodem is ook sterk vervuild met arseen. (24 augustus 1994). *De Tijd*.

Lastenboek Milieu & Gezondheid. Lastenboek voor de aanvraag tot erkenning en betoelaging als steunpunt voor beleidsrelevant onderzoek voor het thema Milieu & Gezondheid. Brussel: Ministerie van de Vlaamse Gemeenschap; 2001

Leroy P, De Geest A: **Milieubeweging en milieubeleid**. Monografieën Leefmilieu Nu; 1985.

Merckx K: **Kris Merckx dokter van het volk**. Berchem: Uitgeverij EPO, 2008.

Sanering ook van wijde omgeving Umicore-fabrieken. (17 januari 2006). *De Standaard*, p. 8.

Thiers G: **Een terugblik op 100 jaar WIV. Lessen uit het verleden en toekomstperspectieven.** Lezing tijdens de Academische Zitting n.a.v. 100 jaar Wetenschappelijk Instituut Volksgezondheid, Brussel, 6 oktober 2004.

Thuwis G. (6 maart 1995): **Rookstop aangeraden voor Noordlimburgers**. *Het Belang van Limburg*, p. 10.

Vandenberghe D, Van Wiele S (February 2, 1999): **Rechter kortgeding: ISVAG moet toch dicht blijven op basis van voorzorgsprincipe**. *Gazet van Antwerpen*, p. 8.

Van Houtte R (January 29, 1999): **De gekleurde wetenschap**. *Gazet van Antwerpen*, p. 7.

Van Wiele S. (24 maart 1998): **Bewoners wijk Neerland boycotten nieuw onderzoek**. *Gazet van Antwerpen*, p.2.

Van Wiele S, Vermeire C (7 november 1997): **Verband ovens en ziekten niet bewezen**. *Gazet van Antwerpen*, p.2.

Vermeire C (10 januari 1998): **Adviescommissie zou oven in Wilrijk willen opstarten**. *Gazet van Antwerpen*, p.7.

Wynants K, Verlaek M: **Medisch milieukundigen bij de LOGO's: Essentiële schakels in de dialoog over milieugerelateerde gezondheidsrisico's**. *Biomonitor* 2004, **2**.
